# ABC Transporters in Human Diseases: Future Directions and Therapeutic Perspectives

**DOI:** 10.3390/ijms23084250

**Published:** 2022-04-12

**Authors:** Thomas Falguières

**Affiliations:** Inserm, Université Paris-Saclay, Physiopathogénèse et Traitement des Maladies du Foie, UMR_S 1193, Hepatinov, 91400 Orsay, France; thomas.falguieres@inserm.fr

The goal of this Special Issue on “ABC Transporters in Human Diseases”, for which I was invited as a Guest Editor, was to provide an overview of the state-of-the-art research, understandings, and advances made in recent years on human diseases implicating ATP-binding cassette (ABC) transporters. Mammalian ABC transporters form a protein superfamily composed of 49 members [1], most of which are transmembrane proteins, responsible for the active transport of their substrates (ions, drugs, peptides, and lipids) across biological membranes, owing to their capacity to bind and hydrolyze adenosine triphosphate (ATP) [2]. Importantly, for approximately a third of these transporters, molecular defects have been correlated with human diseases [3,4], the most famous of which being cystic fibrosis, due to mutations of the chloride channel cystic fibrosis transmembrane conductance regulator (CFTR/ABCC7). Concerning CFTR/ABCC7, in this Special Issue, Uliyakina et al. investigated the contribution of the regulatory extension and regulatory regions—two distinct parts of the regulatory domain of the channel—in the rescue efficiency of the corrector VX-809 and the potentiator VX-770 on the F508del variant [5]. To treat patients affected by these ABC-transporter-related diseases, it is important to (1) understand the molecular mechanisms regulating the expression, intracellular traffic, and activity of these ABC transporters in normal and pathological conditions; (2) characterize the molecular and cellular effects of genetic variations identified in patients that relate to the genes encoding these transporters; (3) find new treatments specifically targeting the previously characterized defects (Figure 1). In this Special Issue, authors made significant contributions by presenting original articles and reviews that cover their fields of expertise on their chosen ABC transporters.

In the field of multidrug resistance (MDR) and cancer, findings of Low et al. suggested that ABCC1 (MRP1) AND ABCC4 (MRP4) are expressed in breast cancer cell lines and that these transporters are implicated in cell proliferation and migration, respectively [6]. Hlaváč et al. proposed that two genetic variations in *ABCC11* and *ABCA13* identified in patients can be associated with breast cancer, even if the data were not found to have strong statistical relevance [7]. Using phylogenic and sequence alignment analyses of the 12 members of the A subfamily ABC transporters, the same research group (Dvorak et al.) identified 13 single nucleotide variations in the 5′-untranslated transcribed region (5′-UTR) of *ABCA* genes in a small cohort of 105 patients with breast cancer [8]. Lower expression of D subfamily ABC transporters, localized at peroxisomes and responsible for very-long fatty acid transport, was also claimed to be involved in the progression of cancer, in addition to the well-known implication of ABCD1 in X-linked adrenoleukodystrophy (X-ALD) pathogenesis, reviewed by Tawbeh et al. [9]. The role of ABCC6, an ATP transporter predominantly expressed in the liver and kidneys, in cancer has also been highlighted, an aspect reviewed by Bisaccia et al. [10]. Thus, targeting this transporter would constitute an alternative anticancer strategy to further investigate. However, ABCC6 defects are mostly known for their implication in the development of pseudoxanthoma elasticum (PXE), a rare and severe disease-causing calcification of soft tissues and mostly characterized by skin lesions and cardiovascular complications, as reviewed by Shimada et al. [11]. As regards ABCC6, Szeri et al. built a new structural model of this transporter based on its homology with bovine Abcc1, which allowed them to highlight the role of specific amino acids in the function of the transporter, using both cell models and 3D analyses [12]. Sasitharan et al. revealed that mutation of the three glutamines into alanines at positions 347, 725, and 990 of ABCB1/MDR1/P-gp, despite their previous implication in taxol binding [13], did not alter taxol transport outside cell models [14].

Addressing the topic of breast cancer resistance protein (BCRP/AGCG2), another ABC transporter also implicated in MDR and gout pathogenesis, Toyoda et al. reported two new genetic variations (M131I and R236X) identified in family members affected by hyperuricemia and gout and responsible for urate transport defects of the transporter [15]. On this topic, Eckenstaler and Benndorf provided a complete review of the literature on the role of ABCG2 in urate homeostasis and its genetic variants involved in the pathogenesis of gout and hyperuricemia [16], while László Homolya proposed a classification of the genetic variants of ABCG2 based on their expression, traffic or function defects, and discussed their implication in human diseases, including cancer [17]. Using phylogenetic, sequence alignment, and structural analyses between members of the G subfamily of human ABC transporters, Mitchell-White et al. identified a conservation pattern that is different in ABCG2, which could confer greater flexibility to this transporter and thus explain its broader range of substrates [18]. Finally, based on already resolved 3D structures of ABCG2 and ABCG5/G8, and in the framework of providing a comprehensive review of the literature and comparative structural analysis, Khunweeraphong and Kuchler proposed a homology model of fungi pleiotropic drug-resistance (PDR) transporters, paving the path to a better understanding of infectious diseases due to pathogenic fungi, thus offering new therapeutic perspectives [19].

Chai et al. described that amyloid-beta peptides accumulating in the brain of patients with Alzheimer’s disease (AD) are substrates of ABCB1/MDR1/P-gp [20], setting the groundwork for alternative therapeutic options. On the same topic of AD, Wanek et al. reported that, in line with former studies in the field, ABCB1/ABCG2 dual substrate drugs were normally distributed in the brain of AD mouse models despite less expression of the two transporters [21]. Evidence is also accumulating on the role of ABCA7 genetic variations in the impaired clearance of amyloid-beta peptides in AD, a topic reviewed by Dib et al. in this Special Issue [22]. It is also interesting to note that ABCA1, a ubiquitous exporter of free cholesterol and phospholipids, has also been implicated in AD pathogenesis, among many other pathologies—namely, type 2 diabetes, infectious diseases, cancer, age-related macular disease, and glaucoma, as reviewed by Jacobo-Albavera et al. [23].

Beyond using cell models and sequence alignments, a striking aspect in the recent body of literature on ABC transporters, including most of the contributions gathered in this Special Issue, is the use of 3D structural analyses to unravel the molecular mechanisms regulating the folding and the function of ABC transporters. Indeed, these approaches are very useful for understanding the cell biology of ABC transporters in normal and pathological conditions, as well as to study the potential direct interactions of small molecules with these transporters. Such approaches are now largely facilitated by the exponentially growing numbers of published 3D structures of ABC transporters in several conformational states (PDB structures available at https://www.rcsb.org/, accessed on 4 April 2022), mostly owing to the recent explosion of cryogenic electron microscopy (cryo-EM), which now allows a resolution below 4 Å, almost making X-ray crystallography outdated [24,25]. As stated recently by Shvarev et al., the next steps for these structural analyses now require the combination of “*biochemical and structural cryo-EM studies with molecular dynamics simulations and other biophysical methods*” [25].

Finally, I would like to focus on canalicular ABC transporters specifically expressed at the canalicular membrane of hepatocytes, in which I am particularly interested in the frame of our research projects. These transporters include ABCB4, ABCB11, and ABCG5/G8 implicated in the secretion of phosphatidylcholine, bile acids, and cholesterol into bile, respectively [26]. Regarding the ABCG5/G8 heterodimer, Williams et al. proposed an interesting review of the literature with a focus on sitosterolemia, a very rare disease caused by genetic variations of these half-transporters [27], while Xavier et al. investigated the effect of three ABCG5/G8 disease-causing missense variations by combining ATP hydrolysis measurements in a cell-free system with molecular dynamics approaches [28]. Sohail et al. provided a review article describing the tools (structures, animals, etc.), allowing the study of ABCB11 pathobiology and exploration of new therapeutic strategies [29]. We also proposed a review article describing the molecular regulation of these canalicular ABC transporters [30], as well as an original study highlighting the role of RAB10 as a new ABCB4 interactor regulating the traffic and function of this transporter [31]. As a therapeutic approach for progressive familial intrahepatic cholestasis (PFIC) implicating genetic variations of canalicular ABC transporters, Bosma et al. described recent advances in AAV8-mediated gene therapy aiming at restoring the expression and function of canalicular ABC transporters, mostly ABCB4, for which defects are implicated in PFIC3, and the limitations of such approaches [32]. Interestingly, an alternative mRNA-delivery-based strategy also aiming at restoring ABCB4 expression and function in mouse models was also recently proposed [33]. However, as stated by Bosma et al. [32], gene therapy cannot be the answer to all PFICs; therefore, pharmacotherapy strategies have to be pursued to propose, in the long run, targeted therapies within the framework of personalized medicine (Figure 1).

## Figures and Tables

**Figure 1:**
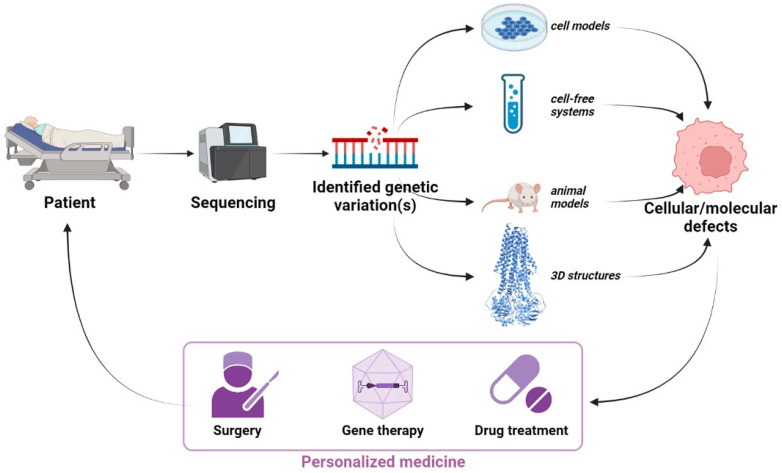
Personalized medicine for ABC-transporter-related diseases. Genes of interest are sequenced from patients to identify genetic variations. Then, the effects of these variations are studied using cell models, cell-free systems, animal models, and 3D modeling, allowing the characterization of the defects induced by the genetic variations. Based on this research, and as a long-term outlook, an adapted strategy for personalized medicine could emerge that would be specific to each patient. Created with BioRender.com.

## Data Availability

Not applicable.
